# Plasma protein binding prediction focusing on residue-level features and circularity of cyclic peptides by deep learning

**DOI:** 10.1093/bioinformatics/btab726

**Published:** 2021-11-22

**Authors:** Jianan Li, Keisuke Yanagisawa, Yasushi Yoshikawa, Masahito Ohue, Yutaka Akiyama

**Affiliations:** Department of Computer Science, School of Computing, Tokyo Institute of Technology, Meguro-ku, Tokyo 152-8550, Japan; AIST-TokyoTech Real World Big-Data Computation Open Innovation Laboratory (RWBC-OIL), National Institute of Advanced Industrial Science and Technology, Tsukuba, Ibaraki 305-8560, Japan; Department of Computer Science, School of Computing, Tokyo Institute of Technology, Meguro-ku, Tokyo 152-8550, Japan; Middle-Molecule IT-based Drug Discovery Laboratory (MIDL), Tokyo Institute of Technology, Kawasaki, Kanagawa 210-0821, Japan; Department of Computer Science, School of Computing, Tokyo Institute of Technology, Meguro-ku, Tokyo 152-8550, Japan; Middle-Molecule IT-based Drug Discovery Laboratory (MIDL), Tokyo Institute of Technology, Kawasaki, Kanagawa 210-0821, Japan; Department of Computer Science, School of Computing, Tokyo Institute of Technology, Meguro-ku, Tokyo 152-8550, Japan; Middle-Molecule IT-based Drug Discovery Laboratory (MIDL), Tokyo Institute of Technology, Kawasaki, Kanagawa 210-0821, Japan; Department of Computer Science, School of Computing, Tokyo Institute of Technology, Meguro-ku, Tokyo 152-8550, Japan; Middle-Molecule IT-based Drug Discovery Laboratory (MIDL), Tokyo Institute of Technology, Kawasaki, Kanagawa 210-0821, Japan

## Abstract

**Motivation:**

In recent years, cyclic peptide drugs have been receiving increasing attention because they can target proteins that are difficult to be tackled by conventional small-molecule drugs or antibody drugs. Plasma protein binding rate (%PPB) is a significant pharmacokinetic property of a compound in drug discovery and design. However, due to structural differences, previous computational prediction methods developed for small-molecule compounds cannot be successfully applied to cyclic peptides, and methods for predicting the PPB rate of cyclic peptides with high accuracy are not yet available.

**Results:**

Cyclic peptides are larger than small molecules, and their local structures have a considerable impact on PPB; thus, molecular descriptors expressing residue-level local features of cyclic peptides, instead of those expressing the entire molecule, as well as the circularity of the cyclic peptides should be considered. Therefore, we developed a prediction method named CycPeptPPB using deep learning that considers both factors. First, the macrocycle ring of cyclic peptides was decomposed residue by residue. The residue-based descriptors were arranged according to the sequence information of the cyclic peptide. Furthermore, the circular data augmentation method was used, and the circular convolution method CyclicConv was devised to express the cyclic structure. CycPeptPPB exhibited excellent performance, with mean absolute error (MAE) of 4.79% and correlation coefficient (*R*) of 0.92 for the public drug dataset, compared to the prediction performance of the existing PPB rate prediction software (MAE=15.08%, R=0.63).

**Availability and implementation:**

The data underlying this article are available in the online supplementary material. The source code of CycPeptPPB is available at https://github.com/akiyamalab/cycpeptppb.

**Supplementary information:**

Supplementary data are available at *Bioinformatics* online.

## 1 Introduction

Conventional small-molecule drugs and antibody drugs cannot interact with 75–80% of potential drug targets (Verdine and Walensky, 2007). Under these circumstances, peptide drug discovery is gaining attention because peptide drugs, unlike conventional small-molecule drugs or antibody drugs, can interact with proteins recruited in important protein–protein interactions (PPIs) (Cary *et al.*, 2017). However, limitations of conventional linear peptides, such as lower stability against degrading enzymes, unsatisfactory selectivity and cell membrane permeability, have not yet been resolved (Cardote and Ciulli, 2016; Vinogradov *et al.*, 2019). In contrast to linear peptides, cyclic peptides have a macrocyclic structure. Macrocyclization imparts a degree of conformational restraint and functional group modification of the cyclized part and contributes to stronger stability and more selective interaction with the target protein (Vinogradov *et al.*, 2019). Therefore, cyclic peptide drugs are thought to improve many pharmacological properties of linear peptides and have favorable properties, such as high target specificity and good potency (Bhat *et al.*, 2015; Cary *et al.*, 2017). Owing to these advantages, more than 40 cyclic peptide drugs were clinically tested, in 2017, and one cyclic peptide drug has been launched to the market per year on average (Zorzi *et al.*, 2017).

However, there remains a challenge related to the pharmacokinetic properties of cyclic peptide drugs (Cary *et al.*, 2017; Vinogradov *et al.*, 2019). Non-ideal pharmacokinetic properties have accounted for about 40% of failures in the traditional drug discovery process (Prentis *et al.*, 1988). Hence, for drug development, it is important to estimate the absorption, distribution, metabolism, excretion and toxicity (ADME-Tox) of compounds at an early stage. Plasma protein binding (PPB) is the reversible binding of compounds to plasma proteins, including serum albumin, hemoglobin and α-acid glycoproteins, and has a substantial effect on the compound ADME-Tox. PPB strongly affects drug distribution and pharmacokinetic behavior with consequences in overall pharmacological action (Lambrinidis *et al.*, 2015). Thus, the rate of binding to plasma proteins (%PPB) for a specific drug is a key indicator for lead optimization. Since experimental determination of %PPB is time consuming, fast computational PPB rate prediction methods are used in the early stage of the drug discovery process for traditional small molecules (Lambrinidis *et al.*, 2015).

Previous PPB rate prediction methods for small molecules can be categorized into the following three approaches: ligand-based methods (Ingle *et al.*, 2016; Sun *et al.*, 2018; Watanabe *et al.*, 2018; Zhu *et al.*, 2013), structure-based methods (Gumede *et al.*, 2012; Katrina *et al.*, 2014) and composite methods that use both ligand and structure-based methods (Chen and Chen, 2012; Li *et al.*, 2011; Zsila *et al.*, 2011). The ligand-based approach uses various molecular descriptors calculated from the ligand molecule structure to establish quantitative structure–activity relationship (QSAR) models. The descriptors of the hydrophobicity index, such as the octanol–water partition coefficient (LogP), are generally the most important features for ligand-based methods (Lambrinidis *et al.*, 2015). For instance, Ingle *et al.* (2016) constructed several machine learning prediction models using 1045 small-molecule drugs and validated 200 independent compounds and 406 environmentally relevant ToxCast chemicals. The best model exhibits prediction accuracy with a mean absolute error (MAE) of 13.3% and coefficient of determination ($R^{2}$) of 0.56.

Nevertheless, since cyclic peptides have a relatively large structure, it is difficult to apply a prediction model for small-molecule drugs directly. Another issue is the limited number of available studies of cyclic peptides. Thus, finding descriptors that can be used for both cyclic peptides and small-molecule compounds has been a major research direction for the computational prediction of the PPB rate of cyclic peptides. Tajimi *et al.* (2018) constructed a prediction model using 1211 experimental data of small molecules, which were collected by Ingle *et al.* (2016), and made PPB rate predictions for the 24 public DrugBank (Wishart *et al.*, 2018) cyclic peptides and 16 in-house cyclic peptides. Since the biophysical mechanism of PPB can be expected to be similar for both small molecules and cyclic peptides, their study focused on selecting descriptors with high generalizability from small-molecule training data. However, the method proposed by Tajimi *et al.* (2018) still has lower prediction accuracy (MAE of 21.6%, correlation coefficient (*R*) of 0.46) than that of the traditional prediction methods for small-molecule compounds and is not accurate enough for practical use. A feasible way to improve prediction accuracy is the consideration of a local structure, such as residue-level features of cyclic peptides. Schneider *et al.* (2017) investigated the structure–activity relationship of daptomycin and its derivatives and found a huge difference in PPB rates of daptomycin and acetyl-daptomycin. This difference is attributed to the tight hydrophobic contacts between the N-terminal fatty acyl chain of daptomycin and human serum albumin (HSA), the main component of the plasma proteins.

Recently, deep learning methods have greatly contributed to several fields, such as speech recognition (Hinton *et al.*, 2012), object recognition (Eitel *et al.*, 2015) and protein tertiary structure prediction (Sato and Ishida, 2019). We thought the convolutional neural network (CNN) model would be suitable for the prediction of the PPB rate of cyclic peptides because it can express the number of residues, sequence information and partial features. In this study, we developed a prediction method called CycPeptPPB based on 1D-CNN that considers findings such as the influence of specific residues on the cyclic peptide PPB mechanism. Moreover, properties such as sequence information and cyclicity of the cyclic peptide were considered. To this end, first, the main chain was divided into residues to represent the partial structure. Using descriptors calculated from these residues, we proposed a 1D-CNN input format that can express the number of residues and sequence position information. In addition, we proposed two methods for expression of the circularity of the cyclic peptide, namely the circular convolution method CyclicConv and data augmentation method.

## 2 Materials and methods

### 2.1 Experimental data

**Fig. 1.**
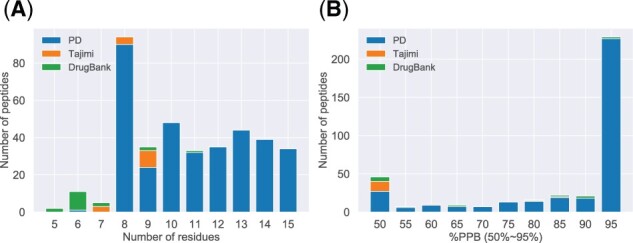
Distributions of experimental data. (**A**) Number of residues. (**B**) Objective variable %$PPB_{50-95}$. Data of ≤50% is included in the leftmost bar, and data of ≥95% is included in the rightmost bar. Blue, orange and green bars indicate PD (PeptiDream), Tajimi and DrugBank datasets, respectively (distribution of the original %*PPB* is also shown in Supplementary Fig. S1)

Studies investigating the PPB of cyclic peptides have not significantly advanced the field, and there are less than 30 peptides available. Our group spent a significant portion of our funding on synthesizing 16 cyclic peptides and conducting PPB rate measurement experiments (Tajimi *et al.*, 2018). However, the PPB rate of these peptides was very low, making it difficult to convert them into actual drug candidate compounds. Therefore, we collaborated with PeptiDream Inc., a leading company in cyclic peptide drug discovery, to provide us with many peptides with excellent PPB rates under a non-disclosure agreement. This allowed us to build a prediction model and to have a scientific discussion. In this study, private data concerning 347 peptides were provided by PeptiDream Inc., and 16 synthesized peptide data, and 17 approved peptide drug data with their experimentally determined PPB rates were used. Figure  1 and Supplementary Figure S1 show the distributions of the number of residues and the experimentally determined PPB rate of each cyclic peptide in each dataset. This section describes these three datasets and how they were split into training and test data.

#### 2.1.1 PeptiDream dataset

The PeptiDream (PD) dataset contained 347 cyclic peptides designed and assayed by PeptiDream Inc. Details of their structural information were confidential; however, many peptides have N-methylated residues, reducing the number of hydrogen bond donors and increasing lipophilicity. A desirable property for drug candidates is %PPB≥80%, and higher lipophilicity tends to enhance PPB rate. Therefore, peptides with %PPB≥80% occupied approximately 80% (272) of the whole dataset.

#### 2.1.2 Tajimi dataset

Tajimi *et al.* (2018) designed 16 cyclic peptides composed only of natural amino acids and conducted %PPB measurement experiments using the equilibrium dialysis method. In this study, these 16 cyclic peptides were used as the Tajimi dataset (Supplementary Table S1). All these cyclic peptides were cyclized by disulfide bonds between the N-terminal and C-terminal cysteine residues. Unlike the PD dataset, the peptides in the Tajimi dataset have not been optimized for enhanced lipophilicity, such as N-methylation. Therefore, these compounds had extremely low %PPB; no compound had more than 90%, and only two compounds had more than 80%.

#### 2.1.3 DrugBank dataset

Tajimi *et al.* (2018) collected 24 cyclic peptide PPB data from the FDA-approved drug public database DrugBank (Wishart *et al.*, 2018). From these data, we extracted 17 cases that were relatively similar in structure to the other two datasets (containing one ring and composed of five residues or more). These 17 public drug data were used as the DrugBank dataset in this study (Supplementary Table S2). The range of %PPB was wide, eight compounds exceeded 80%. Unlike other datasets, some of these cyclic peptides contained fatty acid side chains; such peptides could exhibit high PPB rates even with low lipophilicity. Thus, the dataset was suitable for verifying the generalization performance of the prediction model and was used as external test data.

#### 2.1.4 Splitting training and internal test data

Since the peptides of the DrugBank dataset are structurally diverse and those of the PD and Tajimi datasets were relatively similar in structure, PD and Tajimi datasets were mixed and split into training and internal test data. The Kennard–Stone (KS) algorithm is a method for constructing a subset, which aims to uniformly cover a multidimensional space by maximizing the Euclidean distance between the vectors of the selected sample (Galvao *et al.*, 2005). We utilized the KS algorithm (Algorithm S1) to extract 10% data from the PD and Tajimi datasets (363 in total) as internal test data (37). The remaining data of PD and Tajimi datasets were used as training data (326).

#### 2.1.5 Objective variable

The objective variables used in PPB prediction studies for conventional small-molecule compounds can be broadly divided into two types: the type where the binding ratio is used as it is (Ingle *et al.*, 2016; Sun *et al.*, 2018; Watanabe *et al.*, 2018) and the type where it is used after logarithmic conversion (Li *et al.*, 2011; Zhu *et al.*, 2013). In the case of conventional small-molecule compounds, the PPB rate tends to be extremely high. Thus, logarithmic conversion, which has high resolution in the region of %PPB>90%, is considered suitable for prediction. However, the PPB rate of cyclic peptides is generally lower than that of small-molecule compounds. The prediction method for cyclic peptides should practically focus on peptides with %PPB ranging from 50% to 95%, and logarithmic conversion is not so effective in the range. Cyclic peptides with %PPB less than 50% are very unlikely to be effective as drugs, and there is no need to precisely quantify %PPB less than 50%. Therefore, in this study, an experimentally measured value of %PPB less than 50% was rounded to 50%, and %PPB higher than 95% was rounded to 95% (%$PPB_{50-95}$, Fig.  1B). 

### 2.2 Descriptor design

#### 2.2.1 Division of the main chain of a cyclic peptide into residues

The specific local structure of a cyclic peptide has great influence on the PPB rate (Schneider *et al.*, 2017). Thus, we expressed local structural information by dividing the main chain of a cyclic peptide into units of residues (strictly speaking, it may not match the residue; thus, it is referred to as the substructure hereafter). The division was applied for peptide bonds and disulfide bonds. However, if the peptide bond was simply hydrolyzed, a new hydrogen bond donor may be generated, and the original physicochemical properties of the cyclic peptide may not be expressed. Hence, the selection of the capping functional group is important. When calculating 2D descriptors of the substructures, the cleaved amide group and carboxyl group were methylated (add ${\text{CH}}_{3}$) and converted to an aldehyde group (add H), respectively. On the other hand, when calculating 3D descriptors of them, the cleaved amide group and carboxyl group were capped with ACE (acetyl group) and NME (N-methyl group). This was called ACE-NME protein capping, which is often used for structural stability of the protein during molecular dynamics (MD) simulations (Isegawa *et al.*, 2013; Zuo *et al.*, 2014). When dividing the disulfide bond, hydrogen atoms were added to both sulfur atoms. In addition, to completely express the properties of the local structure, amide bonds existing anywhere other than the macrocycle was not subject to division.

#### 2.2.2 Calculation of descriptors

A total of 323 descriptors consisting of 206 2D descriptors and 117 3D descriptors were calculated by the MOE software (version 2019.01) (Chemical Computing Group Inc., 2019). The details of the 3D descriptors calculation are described in Supplementary Text S1. Descriptors calculated from both the whole peptide (whole-peptide descriptors) and each substructure (substructure descriptors) were standardized using Z-score as $z_{i}=\frac{x_{i}-\mu }{\sigma }$ (*μ* is the average value of whole-peptide descriptor ***x***, and *σ* is the standard deviation of ***x***). In the case of substructure descriptors, each descriptor value for a specific substructure is weighted based on its appearance frequency within the experimental data used (Supplementary Fig. S2). Whole-peptide predictors were used to select descriptors and construct comparison models, and substructure descriptors were used to construct baseline and CycPeptPPB models.

### 2.3 Descriptor selection

#### 2.3.1 Preprocessing of whole-peptide descriptors

To remove the meaningless descriptors for PPB prediction, preprocessing based on whole-peptide descriptors was performed.

1. Whole-peptide descriptors with values that were constant among all cyclic peptides were removed (323 → 294 descriptors).
2. For whole-peptide descriptor pairs with an absolute value of the correlation coefficient of 0.95 or more, the one with a lower correlation with the objective variable %$PPB_{50-95}$ was removed (294 → 147 descriptors).

These 147 whole-peptide descriptors are listed in the Supplementary Data.

#### 2.3.2 Selection of highly interpretable descriptors

The number of descriptors was still large, and further selection of feasible descriptors was needed. Bolasso (Bach, 2008) is a feature selection method that incorporates the bootstrap method into Lasso (Tibshirani, 1996) and can extract highly important feature sets. Thus, whole-peptide descriptors were selected using a method like Bolasso. The feature selection algorithm is described in Algorithm S2. Three descriptors, logP(o/w), PEOE_VSA–1 and logS, were selected as a result. The changes in the selected descriptors due to the Lasso hyperparameters *α* are discussed in Section 4. The same three substructure descriptors were selected to construct baseline and CycPeptPPB models.

### 2.4 Designing prediction techniques suitable for cyclic peptides

#### 2.4.1 Construction of 1D-CNN input feature map by substructure descriptors

First, the main chain of the cyclic peptide was cut at the cyclized position. Next, the substructure descriptors were arranged in the center of the input layer as shown in Supplementary Figure S3 based on the sequence information, to generate the input feature map of the 1D-CNN model. This input method can express the number of substructures and sequence position information of the substructures.

#### 2.4.2 Proposed convolution method (CyclicConv)

**Fig. 2.**
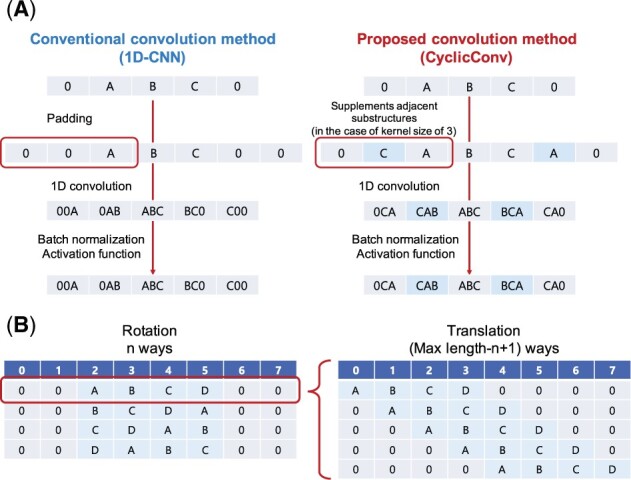
(**A**) Comparison of the conventional convolution method (1D-CNN) and the proposed convolution method (CyclicConv). In the case of kernel size of 3, substructure C is supplemented to the left of A and substructure A is supplemented to the right of C. By this operation, the information of CAB and BCA can be correctly acquired as a result of the CyclicConv. (**B**) Augmentation (rotation and translation) based on the sequence information

The conventional 1D-CNN cannot express circularity. To overcome this limitation, we proposed a new convolution method that supplemented adjacent substructures at both ends of an input peptide sequence (CyclicConv, Fig.  2A).

#### 2.4.3 Augmentation for input feature map

In addition to CyclicConv, we proposed data augmentation as a method to express cyclical structure. As shown in Figure  2B, data augmentation was performed based on the rotation and translation of the input peptide sequence. The peptide with the largest number of substructures in the experimental data was composed of 15 substructures, and augmentation yields n×(Max_length(=15)−n+1) feature map replicas from the cyclic peptide with *n* substructures (363 peptides → 18 999 replicas). In addition, augmentation can express cyclic information of cyclic peptides to some extent. Moreover, predictions can be performed based on relative position information rather than absolute position information of a sequence, leading to improved generalization performance and robustness of the prediction model.

### 2.5 Architecture of prediction models

#### 2.5.1 Baseline model and three CycPeptPPB models

**Fig. 3.**
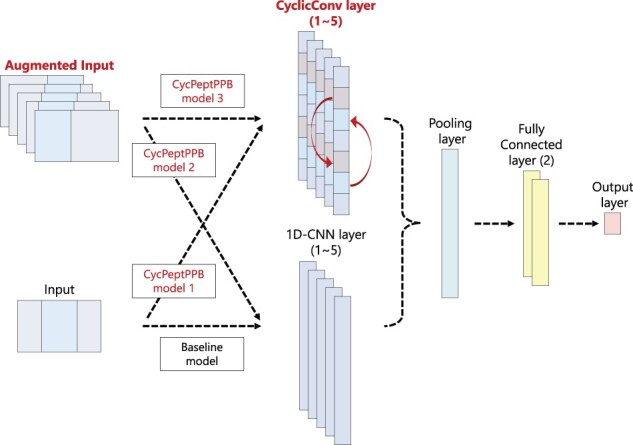
Architecture of the baseline model and CycPeptPPB models 1, 2 and 3. The structure of the pooling layer and the fully connected layer was the same for all four models. In addition, all convolutional layers and fully connected layers used batch normalization

Using the input format of the 1D-CNN model described earlier, we constructed four CNN prediction models (a baseline model and the CycPeptPPB models 1, 2 and 3) based on substructure descriptors. These models were built with the Chainer framework (version 7.1.0) (Tokui *et al.*, 2015). The model using the ordinary convolutional method was used as the baseline model, and the model using CyclicConv was used as CycPeptPPB model 1. Furthermore, to assess the effect of augmentation, we devised CycPeptPPB model 2, which combines augmentation and the ordinary convolution method and CycPeptPPB model 3 that combines augmentation and CyclicConv. Figure  3 shows the basic architecture of these CNN models and differences among them.

Several efficient hyperparameter search methods based on Bayesian optimization have been widely used (Ban *et al.*, 2017). In this study, we used Optuna (version 2.2.0) (Akiba *et al.*, 2019) to optimize hyperparameters, including the number of convolutional layers and CyclicConv layers for each CNN model. The search range of target hyperparameters and their search results are shown in Supplementary Tables S3 and S4.

#### 2.5.2 Comparison with conventional methods

We also compared CycPeptPPB models with three conventional methods to assess the importance of using the substructure descriptor. ADMET Predictor (version 10.0) (Simulations Plus Inc., 2020) is an integrated suite of cheminformatics that examines how the molecular structure of a compound is related to the ADME-Tox properties. To compare the CycPeptPPB model with the existing PPB rate prediction software, we used ADMET Predictor as the comparison model 1. Additionally, an SVM model (comparison model 2) and RF model (comparison model 3) based on three selected whole-peptide descriptors were constructed. The hyperparameters of these two models were determined by grid search. The search range and optimal hyperparameters are shown in Supplementary Table S5.

### 2.6 Evaluation method

Supplementary Figure S4 shows the overall flow of the model prediction accuracy evaluation. When using the data augmentation technique to increase the amount of experimental data, not only training but also test data input was augmented, and the average predicted value of all replicas was used as the final predicted value. MAE (%) and *R* were used as evaluation indices in this study. If the predicted value was less than 50%, it was rounded to 50%, and if it exceeded 95%, it was rounded to 95%. The definitions of these indices are shown in Equations (1) and (2). Here, *y_i_* is the experimental value of the *i*th data, and $\bar{y}$ is the average value of the experimental value. ${\hat{y}}_{i}$ is the predicted value of the *i*th data, and $\bar{\hat{y}}$ is the average value of the predicted values.

(1) $$\text{MAE}=\frac{\sum_{i=1}^{n}\left|y_{i}-{\hat{y}}_{i}\right|}{n}$$

 

(2) $$R=\frac{\sum_{i=1}^{n}\left({\hat{y}}_{i}-\bar{\hat{y}}\right)(y_{i}-\bar{y})}{\sqrt{\sum_{i=1}^{n}{\left({\hat{y}}_{i}-\bar{\hat{y}}\right)}^{2}}\sqrt{\sum_{i=1}^{n}{\left(y_{i}-\bar{y}\right)}^{2}}}$$

From the viewpoint of compound screening, whether the PPB rate exceeded 80% was an important consideration for drug development. In addition, drugs with a high binding rate may differ significantly from drugs with a low binding rate in terms of tissue penetration and half-life (Scheife, 1989). Therefore, to select a better compound, it is important to evaluate whether the PPB rate in the region exceeds 80%. In this study, in addition to the prediction accuracy of %PPB from 50% to 95% (%$PPB_{50-95}$), the prediction accuracy of 80–95% was evaluated ($\%PPB_{80-95}$). In the latter case, cyclic peptides with experimental values of 80% or more were selected as calculation targets from test data. The evaluation indices were calculated from these predicted values. Similarly, if the predicted value exceeded 95%, it was rounded to 95%.

### 2.7 Analysis of prediction results by Saliency Score

Cyclic peptides composed of natural amino acids of *x* residues have $20^{x}$ combinations, and it is computationally impossible to perform a trial-and-error-based search of the entire space for a better PPB rate. Therefore, from the perspective of efficient design and optimization for the development of cyclic peptide drugs, determining which substructure enhances %PPB and which has no effect on %PPB is necessary. We analyzed the importance of each substructure using the Saliency Score defined with the Saliency Map and calculated the contribution of the partial substructure to the PPB rate prediction.

The Saliency Map was originally defined as a heatmap that estimates the parts of a visual image to which people pay attention when viewing it. Many methods have been reported to calculate the Salience Map of deep learning models (Garcia-Diaz *et al.*, 2012; Kümmerer *et al.*, 2014), and VanillaGrad (Baehrens *et al.*, 2010) is one of the simplest methods. We applied it to calculate Saliency Score $s_{i}=(s_{ij})$ for peptides *i* based on the input (feature map) $x_{i}=(x_{ij})$ and output (predicted value) ${\hat{y}}_{i}$ as $s_{ij}=|\partial {\hat{y}}_{i}/\partial x_{ij}|$.

## 3 Results

### 3.1 PPB rate prediction results

**Fig. 4.**
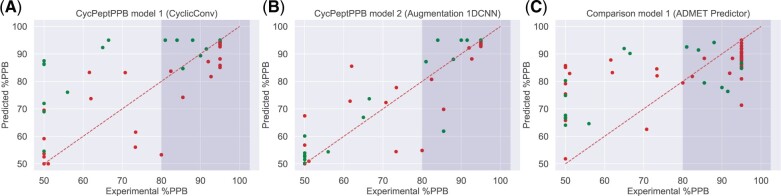
Prediction results of CycPeptPPB model 1 (**A**), model 2 (**B**) and comparison model 1 (**C**), for internal test data (red) and external test data (green). The horizontal axis is experimental %PPB, the vertical axis is predicted %PPB of each method, and the dark background color shows the range over 80%

The prediction accuracy for internal test data and external test (DrugBank) data by all seven models is shown in Tables  1 and 2, respectively. In addition, the experimental %PPB and predicted %PPB for comparison model 1 (ADMET Predictor), CycPeptPPB model 1 (CyclicConv) and CycPeptPPB model 2 (augmentation) are plotted in Figure  4, whereas the results for the other four models are plotted in Supplementary Figure S5. According to the prediction results shown in Table  1 (internal test data), comparison model 1 (ADMET Predictor) exhibited the worst prediction accuracy for test data (R=0.60 in 50–95%). This result showed that the conventional method cannot accurately predict %PPB of cyclic peptides. Comparison model 2 (SVM) and comparison model 3 (RF), using the whole-peptide descriptors calculated from the entire cyclic peptide (R=0.79 through 0.81 in 50–95%), exhibited higher prediction accuracy than the comparison model 1. The results indicated that the proposed whole-peptide descriptor selection method worked correctly and that the three descriptors, logP(o/w), PEOE_VSA-1 and logS, were appropriate for prediction. The baseline model and CycPeptPPB model 1 (CyclicConv), using substructure descriptors, obtained higher prediction accuracy than any of the comparison models (R=0.88 through 0.89 in 50–95%). Both models exhibited similar prediction accuracies, and CyclicConv did not notably improve prediction accuracy compared to the baseline model. CycPeptPPB model 2 (augmentation) and CycPeptPPB model 3 (CyclicConv and augmentation) further improved prediction accuracy compared to the baseline model and CycPeptPPB model 1 (MAE=3.99% through 4.06%, R=0.90 in 50–95%).

**Table 1.** Prediction accuracy of internal test data

%PPB range	Evaluation index	Baseline model	CycPeptPPB model 1	CycPeptPPB model 2	CycPeptPPB model 3	Comparison model 1	Comparison model 2	Comparison model 3
50–95%	MAE (%)	5.09	5.50	3.99	4.06	11.17	6.83	7.86
	*R*	0.89	0.88	0.90	0.90	0.60	0.81	0.79
80–95%	MAE (%)	4.64	3.85	2.65	2.00	6.10	3.65	6.13
	*R*	0.94	0.85	0.93	0.83	0.37	0.59	0.66

*Note*: The best result for each evaluation index is indicated in bold.

**Table 2.** Prediction accuracy of external test data (DrugBank dataset)

%PPB range	Evaluation index	Baseline model	CycPeptPPB model 1	CycPeptPPB model 2	CycPeptPPB model 3	Comparison model 1	Comparison model 2	Comparison model 3
50–95%	MAE (%)	6.55	15.60	4.79	8.97	15.08	13.31	12.55
	*R*	0.89	0.66	0.92	0.87	0.63	0.42	0.50
80–95%	MAE (%)	7.69	4.22	6.17	7.61	9.64	22.92	20.53
	*R*	0.17	0.13	0.43	0.14	–0.41	–0.56	–0.50

*Note*: The best result for each evaluation index is indicated in bold.

For the external test data, in contrast to the internal test data, all comparison models exhibited comparable accuracy in the range of 50–95%, as shown in Table  2. However, since comparison model 1 (ADMET Predictor) tended to predict high PPB rates for most cyclic peptides (about 70% or more, Fig.  4C), the prediction accuracy was higher than comparison model 2 and 3 (SVM and RF, respectively) in the range of 80–95%. As CycPeptPPB model 1 (CyclicConv) also tended to predict high PPB rates for external test data, the MAE was the best among the models in the range of 80–95% (Fig.  4A), whereas there was no correlation in the range of 80–95%. Overall, due to the difficulty in predicting PPB rates of peptides with fatty large side chains in the external test data, the prediction accuracy in the range of 80–95% was inferior to that in 50–95% for all seven prediction models (more detailed discussion was summarized in Supplementary Text S2). CycPeptPPB model 2 (augmentation) exhibited the best performance for the external test data (Fig.  4B, R=0.92 in 50–95%). CycPeptPPB model 3 (CyclicConv and augmentation) was not better than CycPeptPPB model 2. Accordingly, the CycPeptPPB model 2 was used as the representative model of this study.

### 3.2 Important substructures contributing to PPB rate prediction

The top 10 substructures (represented by original #IDs defined in this study) with the highest contributions calculated from the Saliency Score were #100(10), #24(1), #54(1), #103(1), #114(1), #125(1), #121(1), #90(11), #118(1) and #41(14) (numbers in the parentheses indicate the number of cyclic peptides with the corresponding substructure). Among these substructures, #103, #114, #125,#121, and #118 were relatively large substructures (Molecular Weight: 221 through 876) from the DrugBank dataset (these structures are shown in Supplementary Fig. S6). Many of the important substructures had alkyl chains and aromatic rings, while the number of hydrogen bond donors and acceptors were small, indicating that there were many hydrophobic structures. In fact, the standardized logP(o/w) of these substructures, indicating extreme hydrophobicity compared with other substructures (e.g. alanine was –0.48), such as 2.54 for #118 and 1.26 for #125. In contrast, hydrophilic substructures had lower Saliency Scores (e.g. arginine ranked 78 out of all 126 substructures). These results suggest that the presence of the hydrophobic side chain is the primary factor that enhanced the PPB rate. Indeed, HSA, which is most abundant in plasma proteins, has a hydrophobic binding pocket (Lambrinidis *et al.*, 2015). A high binding rate with HSA may be obtained in the presence of a structure that can bind to the hydrophobic binding pocket, such as fatty acids (Schmidt *et al.*, 2013), which is in accordance with our results.

## 4 Discussion

### 4.1 Selected descriptors

In descriptor selection, the hyperparameter *α* of Lasso was changed by 0.2 steps in the range of 0.5 through 4.9. Supplementary Table S6 shows the top seven frequently selected descriptors with varying *α*. Descriptors for lipophilicity (logP(o/w), SlogP etc.), hydrophilicity (logS) and partial charge (PEOE_VSA-1, PEOE_VSA_FPNEG) were often selected regardless of the value of *α*. Lipophilicity-related descriptors are important for PPB rate prediction (Ingle *et al.*, 2016; Lambrinidis *et al.*, 2015). Among them, logP(o/w), the logarithm of the water-octanol partition coefficient, is moderately correlated with the PPB rate (*R* with $\%PPB_{50-95}$ is 0.68). Hydrophobic compounds with a negative charge tend to bind to HSA well. Therefore, partial charge-related descriptors that depend on the ionization state of the peptide, in addition to lipophilicity descriptors, may enhance prediction performance. The relationship between ionization states and prediction accuracy is discussed further in Supplementary Text S3. Most 3D descriptors calculated by MOE were related to potential energy, polar surface area, etc., and no 3D descriptors appeared among the top seven in any hyperparameter *α*. Cyclic peptides in the PD dataset and Tajimi dataset might be described only with 2D descriptors that do not use any 3D structural information.

**Fig. 5.**
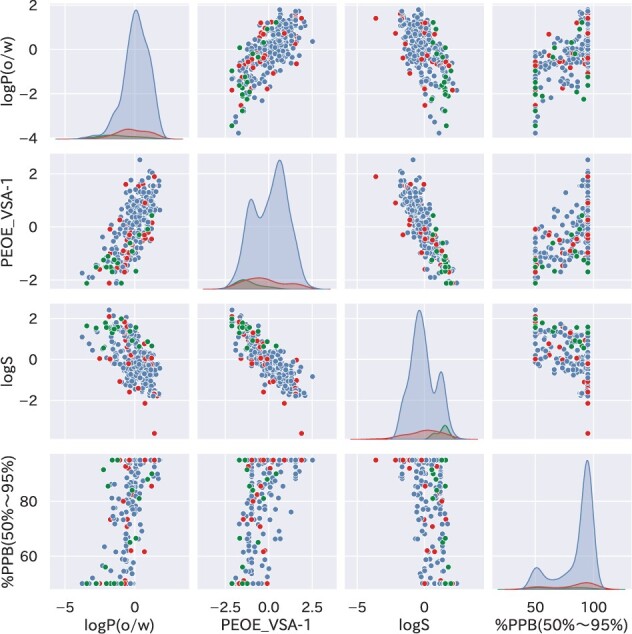
Distribution of three selected whole-peptide descriptors and objective variables. Cyclic peptides in training data, internal test data and external test data (DrugBank dataset) are shown as blue, red and green dots, respectively

With respect to learning time and overfitting, it is desirable to use minimal number of descriptors. Accordingly, three descriptors (logP(o/w), PEOE_VSA-1, logS) consistently selected with the range of α=4.3 through 4.9 were used. The extraction of descriptors facilitated the training of prediction models, although the training data were less extensive than that of general deep learning models. The distribution of the selected whole-peptide descriptors and that of the objective variable are shown in Figure  5, while the distribution of substructure descriptors is shown in Supplementary Figure S7. All three whole-peptide descriptors had a correlation with the objective variable (|R|=0.61 through 0.68). Peptides of the external test data were widely distributed with reference to logP(o/w) but were biased with reference to the other two descriptors. Therefore, it is considered difficult to predict the PPB rate of the external test data with a single descriptor alone.

### 4.2 Principal component analysis revealed the effectiveness of the descriptor selection

**Fig. 6.**
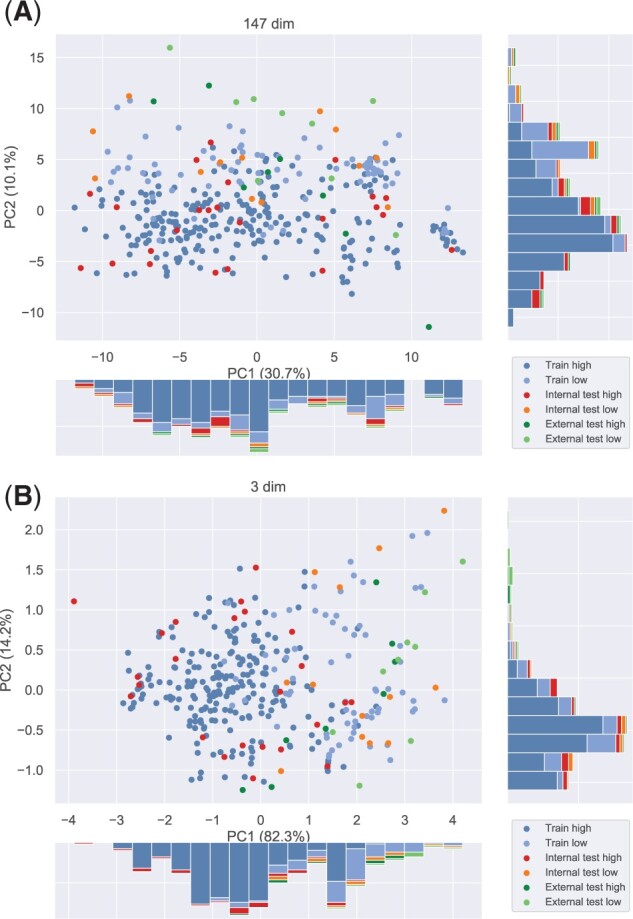
Distribution of each dataset in PCA space, with the first principal component (PC1) as the horizontal axis and the second principal component (PC2) as the vertical axis. The contribution rates are shown in the parentheses of axes captions: (**A**) before descriptor selection (147 descriptors), (**B**) after descriptor selection (three descriptors). High and low indicate data with %PPB≥80% and %*PPB* < 80%, respectively. Blue and light blue indicate training data, red and orange indicate internal test data and green and yellow-green indicate external test (DrugBank) data

To compare the property distributions of internal and external test data relative to the training data, principal component analysis (PCA) was performed. The internal and external test data were converted using the eigenvectors obtained from the training data. From the results shown in Figure  6A, various cyclic peptides spreading in the principal component space were selected as the test data by the KS algorithm. However, the summed contribution rate of both PC1 and PC2 was approximately 40%, and it was difficult to distinguish whether the PPB rate was over 80% or not, based on these two components. Some cyclic peptides from the external test data seemed dissimilar to all peptides of the training data. In contrast, the PCA space with three selected descriptors (Fig.  6B) showed that the external test data were almost in the same range as the training data, despite the unique structures of the cyclic peptides in the external test data. The results indicated that the PPB rate of the external test data can be predicted using selected descriptors. Though it is difficult to distinguish between the high %PPB and low %PPB of external test data with PC1 only, it is possible to distinguish internal test data to some extent. If the PC1 value was less than 0.0, the PPB rate tended to be 80% or higher for training and internal test data. When the value of PC1 exceeded 2.0, the PPB rate tended to be less than 80% for training and internal test data. Thus, the appropriate whole-peptide descriptors were successfully selected by the proposed descriptor selection procedure.

### 4.3 Analysis of acetyl-daptomycin and daptomycin predictions with similar main structures

**Fig. 7.**
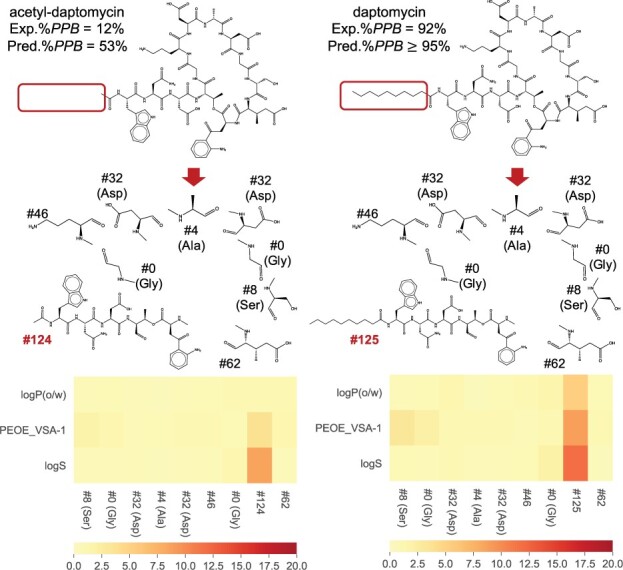
The structures, the substructures obtained by the decomposition procedure, their substructure numbers, and the heatmap of Salience Score of acetyl-daptomycin (Exp.%PPB= 12%, Pred.%PPB= 53%) and daptomycin (Exp.%PPB= 92%, Pred.%PPB≥ 95%). The heatmap of the Salience Score shows the average value of the Salience Score of all augmentation replicas, and the horizontal axis shows the substructure number

The external test (DrugBank) data contained some structurally similar pairs. We analyzed the prediction results of acetyl-daptomycin and daptomycin (Fig.  7) among them, which differed only in the fatty acid side chain corresponding to the substructures #124 and #125. According to the results of Schneider *et al.* (2017), the N-terminal fatty acid side chain of daptomycin (substructure #125) specifically binds to HSA by deeply piercing the binding pocket Site 1, resulting in the high PPB rate (92%). In contrast, acetyl-daptomycin does not have the fatty acid side chain, resulting in the low PPB rate (12%). These differences, however, were hardly reflected to whole-peptide descriptors; thus, Tajimi *et al.* (2018) reported that it was difficult to distinguish them (predicted %PPB value by Tajimi of acetyl-daptomycin: 18%; daptomycin: 49%). The same tendency was obtained with our whole-peptide descriptor-based models (comparison models 2 and 3, predicted $\%PPB_{50-95}$ value of acetyl-daptomycin: 51% through 56%; daptomycin: 53% through 57%).

However, our CycPeptPPB model, which is based on substructure descriptors, correctly predicted their PPB rates. From the heat map of Salience Score shown in Figure  7, both #124 and #125 attracted the most attention from the prediction model, and the model distinguished the structural change even though these two structures were not utilized in model training. The prediction values and analysis suggested that the proposed method with substructure descriptors was effective. It also revealed that the Saliency Score could detect important side chains.

## 5 Conclusion

In this study, a high-performance PPB rate prediction model, CycPeptPPB, for cyclic peptides based on deep learning techniques was proposed. CycPeptPPB model 2, utilizing data augmentation, succeeded in obtaining excellent prediction accuracy (MAE of 4.79%, R of 0.92) for external test data. This model was able to distinguish between the PPB of acetyl-daptomycin and daptomycin, which are similar in structure but differ with respect to PPB rate. Furthermore, we proposed the use of Salience Score as a method for identifying the substructures that are important for predicting PPB rate. The Salience Score can provide insight into actual drug development and support lead compound optimization. Although we provided 3D descriptors as well as 2D descriptors, no 3D descriptor was selected. Future improvements on the usage of 3D conformation information might contribute to better prediction performance. The proposed prediction method allows the selection and design of cyclic peptides with a high PPB rate, which reduces the failure rate in clinical tests and accelerates the development of cyclic peptide drugs.

## Supplementary Material

btab726_supplementary_data
