# Supplementary material for: Plasma protein binding prediction focusing on residue-level features and circularity of cyclic peptides by deep learning
Source: Bioinformatics. 2021 Nov 22;38(4):1110–7. doi: 10.1093/bioinformatics/btab726 (PMC8796384; doi:10.1093/bioinformatics/btab726)
Supplement: btab726_supplementary_data [file btab726_supplementary_data.zip › 20210922_Supplementary_Data.pdf]

Supplementary Data

Plasma protein binding prediction focusing on residue-level features and circularity of cyclic peptides by deep learning

Jianan Li[1,3], Keisuke Yanagisawa[1,2], Yasushi Yoshikawa[1,2], Masahito Ohue[1,2], and Yutaka Akiyama[1,2,*]

[1]Department of Computer Science, School of Computing, Tokyo Institute of Technology, Meguro-ku, Tokyo 152-8550, Japan
[2]Middle-Molecule IT-based Drug Discovery Laboratory (MIDL), Tokyo Institute of Technology, Kawasaki, Kanagawa 210-0821, Japan
[3]AIST-TokyoTech Real World Big-Data Computation Open Innovation Laboratory (RWBC-OIL), National Institute of Advanced Industrial Science and Technology, Tsukuba, Ibaraki 305-8560, Japan

*:To whom correspondence should be addressed.
Contact: akiyama@c.titech.ac.jp

1 Supplemental Texts

Text S1 3D structures of experimental data

S1.1 The 3D structure generation and conformational search

3D structures of substructures and whole-peptides with ionization states at pH 7.0 ± 2.0 were generated using LigPrep software (version 2019.1) [1], and the conformational search was then conducted using MacroModel software (version 2019.1) [2]. The dihedral angle sampling method (Monte Carlo Molecular Mechanics) with enhanced mode was used for the conformational search. To calculate substructure descriptors, the maximum number of searches, energy window, and the cut-off threshold for redundant coordination were set to 1,000, 21.0 kJ/mol, and 0.5 Å, respectively. While calculating whole-peptide descriptors, considering the size of the peptide, the maximum number of searches, energy window, and cut-off threshold for redundant coordination were set to 15,000, 100.0 kJ/mol, and 8.0 Å, respectively.

S1.2 The 3D descriptor calculation

As the values of 3D descriptors depend on the conformation, multiple conformations were generated for their calculation. The representative value of a 3D descriptor was calculated from multiple conformations using the Boltzmann weighted microstate probability. The probability P_i of a conformation i was calculated using the potential energy E_i of the conformation i as shown in Eq.S1.

$$P_i = \frac{1}{Z} \cdot e^{-\beta \cdot E_i} \tag{S1}$$

$$Z = \sum_i e^{-\beta \cdot E_i} \tag{S2}$$

$$\beta = \frac{1}{k_{\mathrm{B}} T} \tag{S3}$$

Here, k_{B} is the Boltzmann constant and T is the temperature parameter. The representative value X was calculated as shown in Eq.S4. Here, x_i is the descriptor x of the conformation i. The temperature parameter T was set to 640.

$$X = \sum_i P_i x_i \tag{S4}$$

Text S2 Predicted results of peptides with PPB rates exceed 80% in the DrugBank dataset

The external test data (DrugBank dataset) contains eight peptides with PPB rates over 80%. The predicted values of these eight peptides obtained from each prediction model were summarized in the Table S7. As can be seen from the MAE of the upper four compounds, comparison model 2 and comparison model 3, which used the whole-peptide descriptors,

were able to obtain relatively high prediction accuracy, like the four CNN models using the substructure descriptors. On the other hand, the prediction results of comparison model 2 and comparison model 3 were very poor for the four compounds in the lower row, which was significantly different from the four CNN models. This result suggests that conventional predictions based on the whole-peptide descriptors may be unable to correctly predict the PPB rate of cyclic peptides with large fatty acid side chain. In addition, our representative model, CycPeptPPB model 2, could not accurately predict the PPB rate of Polymyxin B, which indicates that the prediction accuracy of 80% to 95% of external test data was poor.

Text S3 Relationship between ionization states and prediction accuracy

The ionization state influenced many descriptors, and investigation of the relationship between prediction performance and ionization state may reveal the applicable compound range of the prediction model. We separated cyclic peptides into four groups according to the ionization states: 100 acidic peptides, 107 basic peptides, 106 neutral peptides, and 67 zwitterionic peptides. Figure S8 shows the ratio of the ionization state in each PPB rate. Some previous studies [3, 4] found that acidic small-molecule compounds tend to have higher PPB rates than basic and neutral compounds because they have a high affinity for plasma proteins. However, no such tendency was observed in this study.
In addition, we analyzed the accuracy of our CycPeptPPB model for each ionization state in detail. First, all cyclic peptides were classified into three classes, namely, Low ($\%PPB < 80\%$), Mid ($80\% \leq \%PPB < 90\%$), and High ($90\% \leq \%PPB$). Table S8 shows the prediction accuracy (MAE) at different binding rate regions in each ionization state. As with most PPB rate prediction studies, the prediction accuracy for cyclic peptides belonging to the High group was better than that for Mid and Low group peptides. CycPeptPPB models 2 and 3 obtained high prediction accuracy for all ionization states. In particular, neutral peptides were accurately predicted (MAE = 0.84% through 3.66%).

2 Supplemental Algorithms

Algorithm S1 Details of the KS algorithm used in this study

Input:

k dimensions whole-peptide descriptors: $\boldsymbol{X} = (\boldsymbol{x}_1, \boldsymbol{x}_2, ..., \boldsymbol{x}_k)$
Total number of PD and Tajimi dataset: N
Number of data in the internal test data: n
Initial internal test data: $\boldsymbol{T} = \{\}$

Calculate the average $\bar{\boldsymbol{X}} = (\bar{x}_1, \bar{x}_2, ..., \bar{x}_k)$ of descriptors.
for $i = 1$ to N **do**
    Calculate the Euclidean distance $D[i]$ between the sample i and the average $\bar{\boldsymbol{X}}$.
end for
Select the sample i with the largest Euclidean distance from the average $\bar{\boldsymbol{X}}$ and put it into $\boldsymbol{T}$.
while number of elements t of $\boldsymbol{T}$ is less than n **do**
    for $i = 1$ to $N - t$ **do**
        for $j = 1$ to t **do**
            Calculate the Euclidean distance $D[i][j]$ between the sample i that has not been selected yet and the sample j of $\boldsymbol{T}$.
        end for
        Calculate the minimum value $\hat{D}[i]$ of $D[i][1]$-$D[i][t]$.
    end for
    Calculate the maximum value of $\hat{D}[1]$-$\hat{D}[N - t]$ and put the sample i into $\boldsymbol{T}$.
end while

Algorithm S2 Details of the algorithm for whole-peptide descriptor selection

Input:

whole-peptide descriptors: $\boldsymbol{X}$
objective variable: $\boldsymbol{Y}$
number of cross validations: k
number of bootstrap replicates: m
appearance threshold: t ▷ k was set to 9, m was set to 20, and t was set to 40

```
Split data (X, Y) for k-fold cross validation.
for i = 1 to k do
    Select train data (X_i, Y_i).
    for j = 1 to m do
        Choose a random n from the range of 40–320.    ▷ n is the size of one replica dataset
        Allow duplicates and randomly select n data (X_ij, Y_ij) from (X_i, Y_i).
        Perform Lasso estimate ŵ_ij for data (X_ij, Y_ij).
        Calculate signal S_ij = {s, ŵ^s_ij ≠ 0}.
    end for
end for
Compute sum S = Σ_i^k Σ_j^m S_ij.
Choose signal above threshold S = {s, s ≥ t}.
Select ŵ^S from (X^S, Y).
```

In mathematical notation: line 1 splits $(\boldsymbol{X}, \boldsymbol{Y})$; line 3 selects $(\boldsymbol{X}_i, \boldsymbol{Y}_i)$; line 6 selects $(\boldsymbol{X}_{ij}, \boldsymbol{Y}_{ij})$ from $(\boldsymbol{X}_i, \boldsymbol{Y}_i)$; line 7 computes $\hat{\boldsymbol{w}}_{ij}$; line 8: $\boldsymbol{S}_{ij} = \{s, \hat{w}_{ij}^{s} \neq 0\}$; line 11: $\boldsymbol{S} = \sum_i^k \sum_j^m \boldsymbol{S}_{ij}$; line 12: $\boldsymbol{S} = \{s, s \geq t\}$; line 13: select $\hat{\boldsymbol{w}}^S$ from $(\boldsymbol{X}^S, \boldsymbol{Y})$.

3 Supplemental Tables

Table S1: Amino acid sequence and experimental value of %*PPB* of the Tajimi dataset

Peptide	Amino acid sequences									%*PPB*
Pep.1	Cys	Tyr	Phe	Gln	Asn	Pro	Arg	Gly	Cys	24.2
Pep.2	Cys	Tyr	Ile	Gln	Asn	Pro	Leu	Gly	Cys	0.5
Pep.3	Cys	Ala	Trp	Lys	Val	Thr	Cys			0.04
Pep.4	Cys	Phe	Pro	Phe	Trp	Lys	Tyr	Cys		61.6
Pep.5	Cys	Trp	Arg	Pro	Arg	Val	Ala	Arg	Cys	0.0
Pep.6	Cys	Phe	Phe	Trp	Lys	Thr	Thr	Cys		26.3
Pep.7	Cys	Lys	Leu	Leu	Lys	Lys	Thr	Cys		0.0
Pep.8	Cys	Tyr	Tyr	Tyr	Tyr	Tyr	Tyr	Tyr	Cys	85.5
Pep.9	Cys	Ala	Gly	Leu	Val	Leu	Ala	Ala	Cys	0.0
Pep.10	Cys	Trp	Val	His	Pro	Gln	Phe	Glu	Cys	36.7
Pep.11	Cys	Asn	Gln	Pro	Trp	Gln	Cys			0.0
Pep.12	Cys	Ser	Phe	Asp	Asp	Trp	Leu	Ala	Cys	80.0
Pep.13	Cys	Tyr	Leu	Ala	Glu	Tyr	His	Gly	Cys	34.9
Pep.14	Cys	Ala	Pro	Ala	Trp	Ala	His	Gly	Cys	7.4
Pep.15	Cys	Phe	Val	Tyr	Ser	Ala	Val	Cys		15.3
Pep.16	Cys	Arg	Ile	Lys	Arg	Tyr	Cys			15.1

Table S2: Prior research description %*PPB* and our survey %*PPB* of the DrugBank dataset (01/02/2021 accessed). If multiple %*PPB* values are listed, the average value was used, and the original range is shown in the parentheses.

Peptide	Tajimi *et al.* [5] description value	%*PPB* in this study	Survey source
Acetyl-Daptomycin	12%	12%	Schneider *et al.* [6]
Anidulafungin	99%	84%	DrugBank [7]
Caspofungin	97%	97%	DrugBank
Colistin	90.4%〜92.9%	56% (55%〜57%)	Couet *et al.* [8]
Cyclosporine	90%	90%	DrugBank
Daptomycin	85%	91.5% (90%〜93%)	DrugBank
Desmopressin	50%	17.3% (15.8%〜18.8%)	DrugBank
Eptifibatide	25%	25%	DrugBank
Lanreotide	79%〜83%	81% (79%〜83%)	Medscape
Micafungin	99%	99%	DrugBank
Octreotide	65%	65%	DrugBank
Oxytocin	30%	30%	DrugBank
Pasireotide	88%	88%	DrugBank
Polymyxin B	55.9%	85.5% (79%〜92%)	DrugBank
Quinupristin	55%〜78%	66.5% (55%〜78%)	Bearden [9]
Terlipressin	30%	30%	DrugBank
Vasopressin	1%	1%	DrugBank

Table S3: Hyperparameter search ranges of the baseline model and CycPeptPPB models 1, 2, and 3. The epoch number was set to 20, the loss function used the mean square error (MSE), the filter size of all convolution layers was set to 3, and the filter size of the Pooling layer was set to 2. Furthermore, the number of search trials by Optuna was set to 200. In addition, 200 trials of the search were performed three times (total 600 trials) to avoid fixing the search range at the start of the search.

Hyperparameter	Search range
Activation function	ReLU, Leaky_ReLU, Swish [10]
Optimizer	Adabound [11], RMSprop
Batch size (baseline model & CycPeptPPB model 1)	$20, 30, ..., 100$
Batch size (CycPeptPPB model $2, 3$)	$400, 600, ..., 3400$
Weight decay rate	10^{-5}–10^{-3} (log-scaled)
Number of convolutional layers	1–5
Number of neurons in each convolutional layer	32–256
Padding size of convolutional layer (baseline model & CycPeptPPB model 2)	$0, 1$
Padding size of convolutional layer (CycPeptPPB model $1, 3$)	0
Pooling layer	Max pooling, Average pooling
Number of neurons in each fully connected layer	32–256

Table S4: Results of the hyperparameter search for the baseline model and CycPeptPPB models 1, 2, and 3

Hyperparameter	Baseline model	CycPeptPPB model 1	CycPeptPPB model 2	CycPeptPPB model 3
Activation function	Swish	Swish	ReLU	Leaky_ReLU
Optimizer	RMSprop	Adabound	Adabound	Adabound
Batch size	50	40	2200	1600
Weight decay rate	2.01×10^{-4}	1.93×10^{-5}	6.32×10^{-5}	6.66×10^{-5}
Number of convolutional layers	3	4	5	5
Number of neurons in each convolutional layer	$[216, 246, 197]$	$[204, 252, 118, 218]$	$[191, 222, 246, 42, 205]$	$[107, 169, 201, 130, 182]$
Padding size of convolutional layer	0	0	1	0
Pooling layer	Average pooling	Max pooling	Max pooling	Max pooling
Number of neurons in each fully connected layer	$[185, 128]$	$[200, 32]$	$[151, 50]$	$[100, 42]$

Table S5: Hyperparameters search range of comparison model 2 (SVM), 3 (RF). The SVM model used the Gaussian (rbf) kernel as a kernel function. Due to the results of a thorough grid search, the penalty parameter C and the kernel coefficient γ of the Gaussian kernel were set to 64 and 0.1, respectively. The number of decision trees n for bagging and the maximum depth of each decision tree max_depth were set to 300 and 5 for the RF model, respectively.

Model	Hyperparameter	Search range
Comparison model 2 (SVM)	C	$2^{-6}, 2^{-5}, 2^{-4}, 2^{-3}, 2^{-2}, 2^{-1}, 2^{0}, 2^{1}, 2^{2}, 2^{3}, 2^{4}, 2^{5}, 2^{6}, 2^{7}$
	γ	$0.005, 0.0075, 0.01, 0.025, 0.05, 0.075, 0.1, 0.25, 0.5, 0.75, 1$
Comparison model 3 (RF)	n	$10, 20, 50, 100, 150, 200, 250, 300, 350, 400, 450, 500, 700, 1000$
	max_depth	$1, 2, 5, 10, 15, 20$

Table S6: Top seven whole-peptide descriptors that most frequently appeared in the choice by Bolasso with specified descriptors. α is the regularization hyperparameter of the Lasso algorithm.

39 descriptors ($\alpha = 0.5$)	21 descriptors ($\alpha = 1.3$)	9 descriptors ($\alpha = 2.1$–2.5)	6 descriptors ($\alpha = 3.5$–3.7)	3 descriptors ($\alpha = 4.3$–4.9)
logS	PEOE_VSA-1	logP(o/w)	logP(o/w)	logP(o/w)
PEOE_VSA_FPNEG	logS	PEOE_VSA-1	PEOE_VSA-1	PEOE_VSA-1
PEOE_VSA-0	logP(o/w)	logS	logS	logS
GCUT_SLOGP_2	reactive	PEOE_VSA_FPNEG	PEOE_VSA_FPNEG	
reactive	SlogP	reactive	reactive	
BCUT_SLOGP_3	PEOE_VSA_FPNEG	SlogP	SlogP	
ast_violation	BCUT_SLOGP_3	BCUT_SLOGP_3		

Table S7: $\%PPB_{50-95}$, logP(o/w) (lipophilicity), and the predicted value obtained from each prediction model of eight peptides with $\%PPB \geq 80\%$ in the DrugBank dataset. The upper four peptides have logP(o/w) higher than 0. The lower four peptides have logP(o/w) less than 0, but all have relatively large side chains, with a fatty acid side chain at the tip. MAE was calculated from four peptides each in the upper and lower rows

Peptide	$\%PPB_{50-95}$	logP(o/w)	Baseline model	CycPeptPPB model 1	CycPeptPPB model 2	CycPeptPPB model 3	Comparison model 1	Comparison model 2	Comparison model 3
Lanreotide	81%	2.59	91.62%	95.00%	87.17%	92.69%	92.53%	92.49%	92.74%
Anidulafungin	84%	1.09	90.37%	95.00%	95.00%	95.00%	91.42%	81.66%	76.08%
Pasireotide	88%	4.39	94.36%	95.00%	88.03%	92.93%	94.16%	95.00%	94.22%
Cyclosporine	90%	5.29	91.37%	89.35%	95.00%	95.00%	77.77%	95.00%	94.55%
MAE	-	-	6.18%	8.16%	5.55%	2.51%	4.62%	5.38%	8.10%
Polymyxin B	85.5%	-4.05	67.20%	84.65%	61.86%	67.11%	79.45%	51.43%	56.95%
Daptomycin	91.5%	-5.03	77.73%	91.78%	95.00%	89.58%	76.34%	53.46%	57.33%
Caspofungin	95%	-2.03	92.48%	95.00%	95.00%	92.26%	84.74%	54.58%	62.17%
Micafungin	95%	-3.17	92.74%	95.00%	95.00%	89.77%	86.66%	50.00%	56.74%
MAE	-	-	9.21%	0.28%	6.78%	6.93%	9.81%	39.24%	23.5%

Table S8: Prediction accuracy (MAE) in each ionization state at different binding rate regions (bold is the best result of each status), calculated by combining the prediction results of nine-fold cross-validation of training data, internal test data, and external test data.

Ionization state	PPB rate range	Number of peptides	Baseline model (1D-CNN)	CycPeptPPB model 1 (CyclicConv)	CycPeptPPB model 2 (Augment 1D-CNN)	CycPeptPPB model 3 (Augment CyclicConv)
Acid	Low	30	6.53%	5.35%	5.59%	**4.64%**
	Mid	11	5.14%	7.44%	6.22%	**3.31%**
	High	59	2.15%	2.02%	**0.93%**	0.98%
Base	Low	20	6.21%	13.84%	**4.10%**	4.96%
	Mid	7	10.02%	6.94%	6.39%	7.72%
	High	80	1.93%	2.84%	1.57%	**0.98%**
Neutral	Low	17	6.79%	3.91%	2.48%	**2.46%**
	Mid	13	5.10%	5.31%	3.66%	3.53%
	High	76	2.45%	2.23%	1.34%	**0.84%**
Zwitterion	Low	31	4.75%	4.66%	**2.79%**	4.03%
	Mid	9	8.66%	6.67%	7.14%	4.37%
	High	27	3.30%	2.80%	2.51%	**1.54%**

Ionization state	PPB rate range	Number of peptides	Comparison model 1 (ADMET Predictor)	Comparison model 2 (SVM)	Comparison model 3 (RF)
Acid	Low	30	27.73%	10.77%	9.14%
	Mid	11	5.68%	6.90%	8.99%
	High	59	3.33%	2.40%	3.96%
Base	Low	20	12.67%	8.97%	9.09%
	Mid	7	**5.46%**	13.47%	14.26%
	High	80	10.58%	2.29%	3.80%
Neutral	Low	17	24.72%	11.22%	13.04%
	Mid	13	4.63%	**3.33%**	4.42%
	High	76	5.47%	2.54%	3.18%
Zwitterion	Low	31	18.19%	16.08%	14.84%
	Mid	9	**1.68%**	8.09%	6.86%
	High	27	8.31%	2.38%	3.81%

4 Supplemental Figures

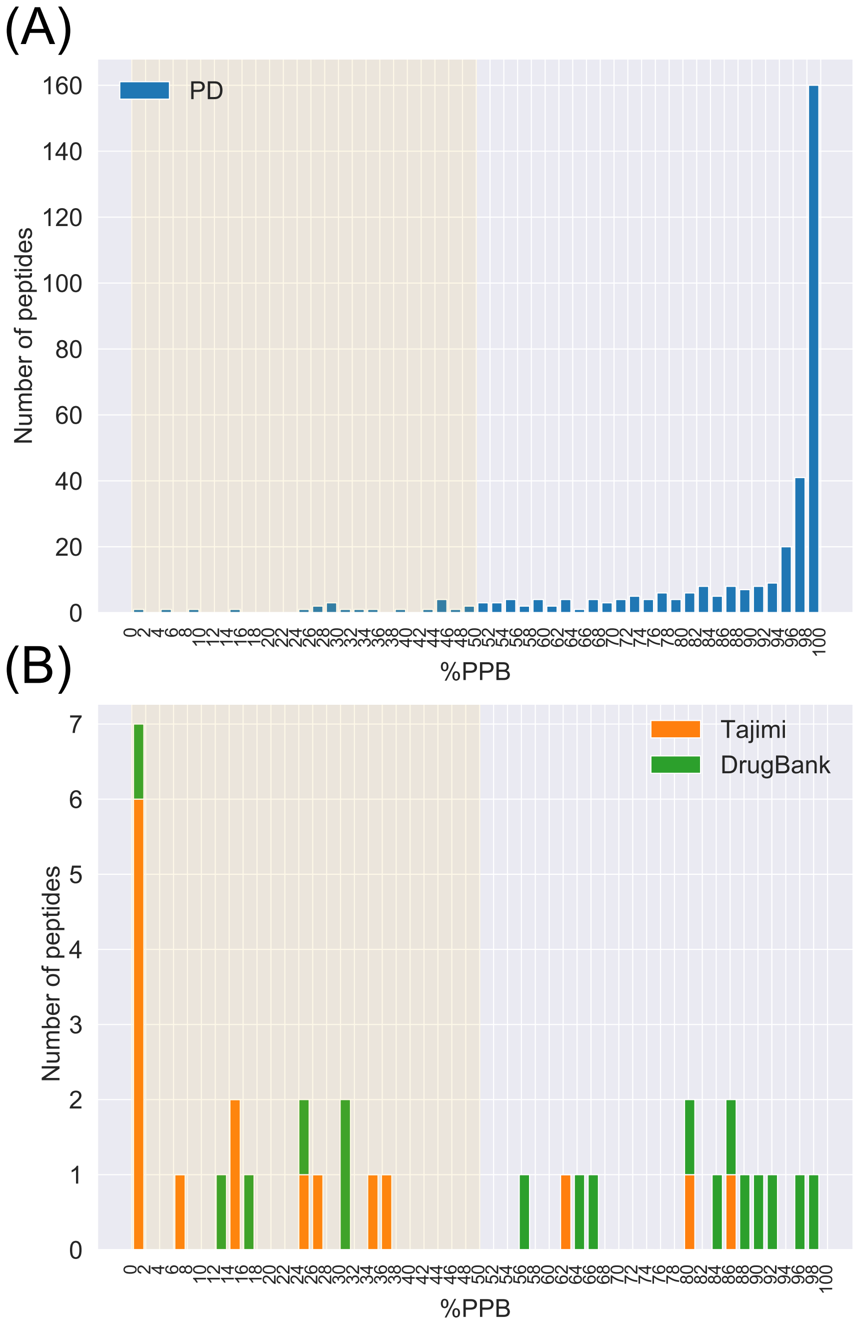

Fig. S1: The distributions of original $\%PPB$ of PD dataset (A), Tajimi dataset and DrugBank dataset (B). The yellow background color shows the range from 0% to 50%.

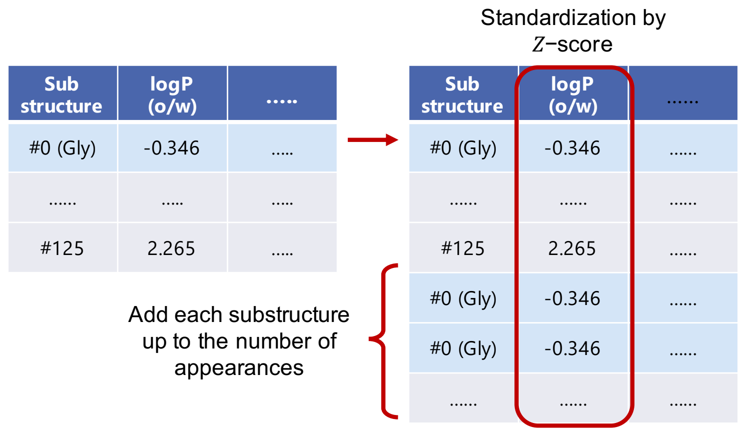

Fig. S2: Substructure descriptor standardization based on the frequency

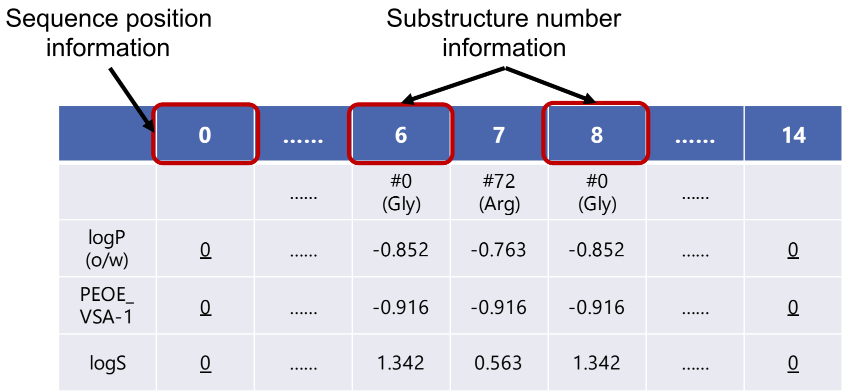

Fig. S3: Input feature map of the 1D-CNN model. For cyclic peptides with an even number of substructures ($2m$), the (m+1)-th substructure is the center. For peptides with less than 15 substructures, the left and right blank areas were padded with 0 to express the difference for the number of substructures.

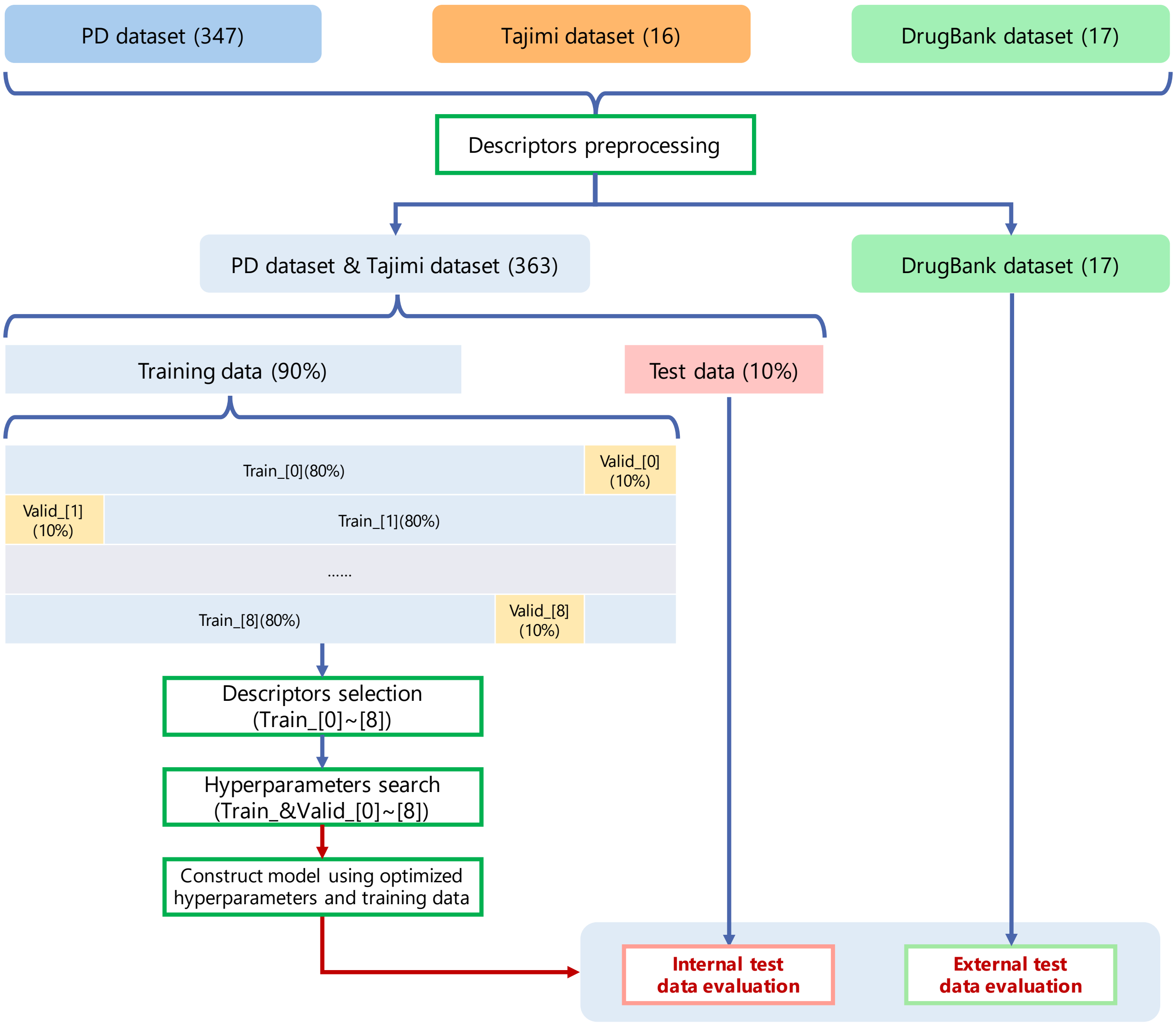

Fig. S4: Overall flow of the construction prediction model from three types of datasets, and prediction accuracy evaluation. Blue arrows and parentheses indicate the flow of data, and red indicates the flow of the prediction model.

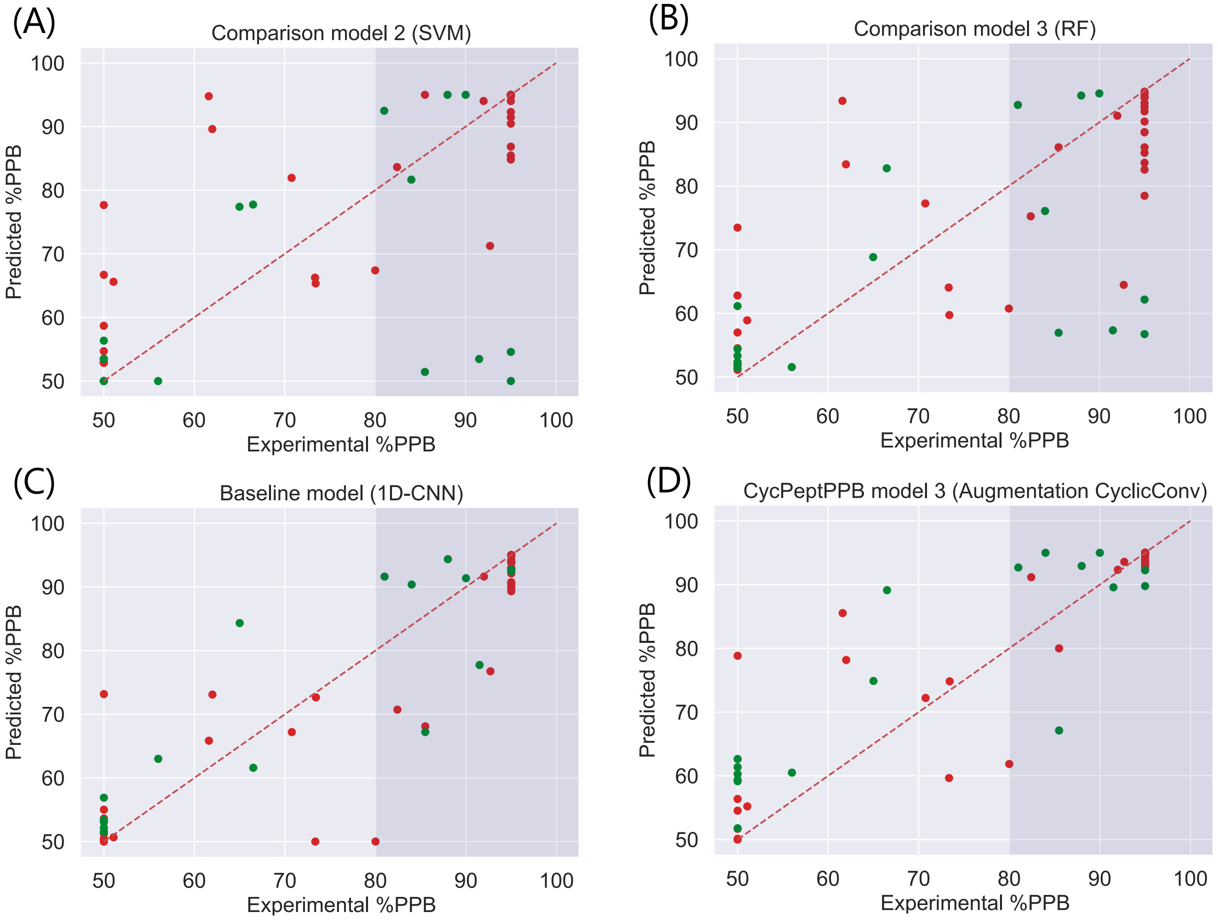

Fig. S5: Prediction results of comparison models 2 and 3 (A, B), baseline model (C), and CycPeptPPB model 3 (D) for internal test data (red) and external test data (green). The horizontal axis is experimental $\%PPB$, vertical axis is predicted $\%PPB$ of each method, and the dark background color shows the range over 80%.

Substructure Number	Structure	Saliency Score (rank)
#103	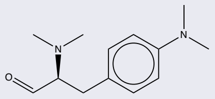	29.85 (4)
#114	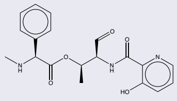	29.07 (5)
#118	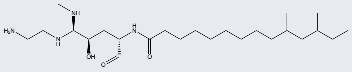	24.00 (9)
#121	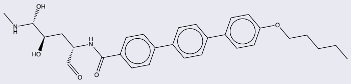	25.67 (7)
#125	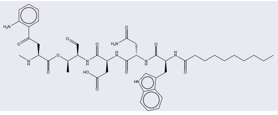	26.02 (6)

Fig. S6: Substructures contained in the DrugBank dataset among the top 10 high contributing substructures

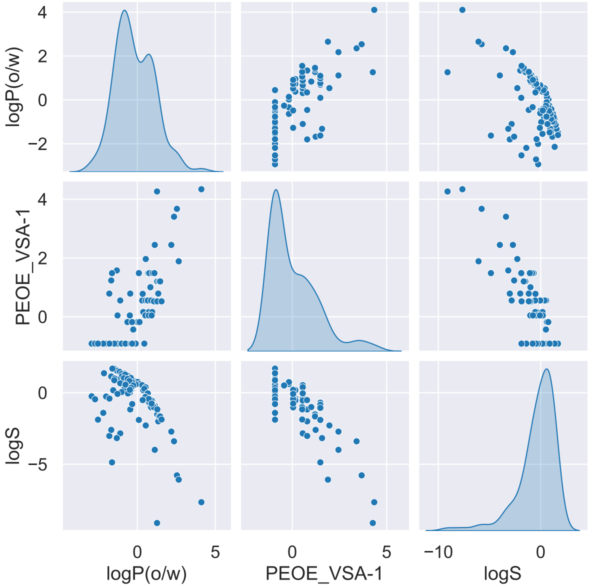

Fig. S7: Distribution of three selected substructure descriptors

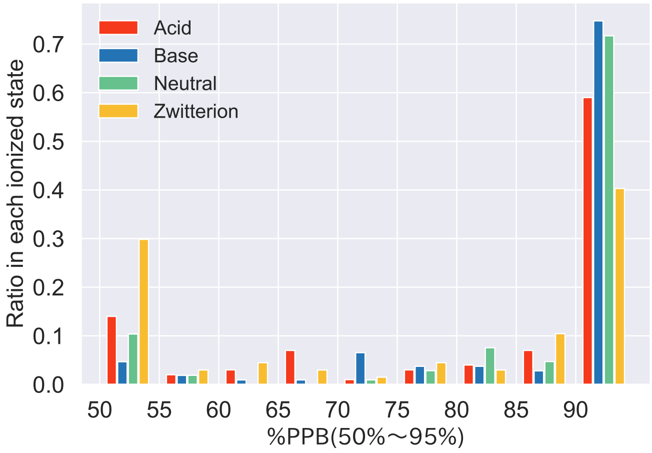

Fig. S8: Ratio of the ionization state in each PPB rate range. All data were binned to every 5% of PPB rate.

References

[1] Schrödinger LLC. (2019a). *Schrödinger Release 2019-1: LigPrep*. New York, NY, USA.

[2] Schrödinger LLC. (2019b). *Schrödinger Release 2019-1: MacroModel*. New York, NY, USA.

[3] Yang, Y., Engkvist, O., Llinàs, A., and Chen, H. (2012). Beyond size, ionization state, and lipophilicity: Influence of molecular topology on absorption, distribution, metabolism, excretion, and toxicity for druglike compounds. *J. Med. Chem.*, **55**(8), 3667–3677.

[4] Watanabe, R., Esaki, T., Kawashima, H., Natsume-Kitatani, Y., Nagao, C., Ohashi, R., and Mizuguchi, K. (2018). Predicting fraction unbound in human plasma from chemical structure: Improved accuracy in the low value ranges. *Mol. Pharm.*, **15**(11), 5302–5311.

[5] Tajimi, T., Wakui, N., Yanagisawa, K., Yoshikawa, Y., Ohue, M., and Akiyama, Y. (2018). Computational prediction of plasma protein binding of cyclic peptides from small molecule experimental data using sparse modeling techniques. *BMC Bioinform.*, **19**(19), 527.

[6] Schneider, E. K., Huang, J. X., Carbone, V., Han, M., Zhu, Y., Nang, S., Khoo, K. K., Mak, J., Cooper, M. A., Li, J., and Velkov, T. (2017). Plasma protein binding structure-activity relationships related to the n-terminus of daptomycin. *ACS Infect. Dis.*, **3**(3), 249–258.

[7] Wishart, D. S., Feunang, Y. D., Guo, A. C., Lo, E. J., Marcu, A., Grant, J. R., Sajed, T., Johnson, D., Li, C., Sayeeda, Z., Assempour, N., Iynkkaran, I., Liu, Y., Maciejewski, A., Gale, N., Wilson, A., Chin, L., Cummings, R., Le, D., Pon, A., Knox, C., and Wilson, M. (2017). DrugBank 5.0: a major update to the DrugBank database for 2018. *Nucleic Acids Res.*, **46**(D1), D1074–D1082.

[8] Couet, W., Grégoire, N., Marchand, S., and Mimoz, O. (2012). Colistin pharmacokinetics: the fog is lifting. *Clin. Microbiol. Infect.*, **18**(1), 30–39.

[9] Bearden, D. T. (2004). Clinical pharmacokinetics of quinupristin/dalfopristin. *Clin. Pharmacokinet*, **43**(4), 239–252.

[10] Ramachandran, P., Zoph, B., and Le, Q. V. (2017). Searching for activation functions. *arXiv preprint*, arXiv:1710.05941.

[11] Luo, L., Xiong, Y., Liu, Y., and Sun, X. (2019). Adaptive gradient methods with dynamic bound of learning rate. In *Proc. ICLR2019*, New Orleans, LA, USA.
